# Analysis of Frequency Drift of Silicon MEMS Resonator with Temperature

**DOI:** 10.3390/mi12010026

**Published:** 2020-12-29

**Authors:** Bo Jiang, Shenhu Huang, Jing Zhang, Yan Su

**Affiliations:** School of Mechanical Engineering, Nanjing University of Science and Technology, Nanjing 210094, China; bjiang@njust.edu.cn (B.J.); huge@njust.edu.cn (S.H.); zhangjing@njust.edu.cn (J.Z.)

**Keywords:** MEMS resonator, temperature coefficient, thermal stress

## Abstract

High-quality-factor Micro-Electro-Mechanical System (MEMS) resonators have been widely used in sensors and actuators to obtain great mechanical sensitivity. The frequency drift of resonator with temperature is a problem encountered practically. The paper focuses on the resonator frequency distribution law in the temperature range of—40 to 60 °C. The four-layer models were established to analyze thermal stress caused by temperature due to the mismatch of thermal expansion coefficients. The temperature variation leads to the transformation of stress, which leads to the shift of resonance frequency. The paper analyzes the influence of hard and soft adhesive package on the temperature coefficient of frequency. The resonant accelerometer was employed for the frequency measurements in the paper. In experiments, three types of adhesive dispensing patterns were implemented. The results are consistent with the simulation well. The optimal packaging method achieves −24.1 ppm/°C to −30.2 ppm/°C temperature coefficient of the resonator in the whole temperature range, close to the intrinsic property of silicon (−31 ppm).

## 1. Introduction

Silicon capacitive resonator, as a primary motion unit, has been widely used in Micro-Electro-Mechanical System (MEMS) accelerometers [1,2], gyroscopes [3,4], pressure sensors [5], and microphones [6]. The capacitive resonator is advantageous to realize displacement amplification under the same driving force, which is suitable for applying MEMS sensors. Silicon material, with high Young’s modulus in the micron scale, is an ideal elastic material. However, the silicon resonator frequency changes with temperature due to the material softening of silicon [7,8]. The temperature coefficient of frequency characterizes the thermal frequency stability of resonators. Different ambient temperature leads to different temperature drift coefficient, affecting the device performance. Many works have been done to improve the temperature performance of the resonator [9,10]. An electronically temperature compensated oscillator based on capacitive silicon micromechanical resonators was implemented to overcome the temperature issues [11]. The oscillator exhibits a frequency drift of 39 ppm over 100 °C as compared to uncompensated frequency drift of 2830 ppm over the same range. Some other works focus on presenting novel structures enlarging the tuning frequency range for temperature drift compensation [12,13,14]. Among them, compensation with different materials or additional static-electrical stiffness was mentioned. The composite resonator with a linear temperature coefficient of frequency was fabricated utilizing silicon and silicon dioxide’s opposing temperature coefficients of Young’s modulus [15]. The temperature-dependent static-electrical forces were applied to reduce the temperature coefficients of polysilicon micromechanical resonators [16].

**Figure 1:**
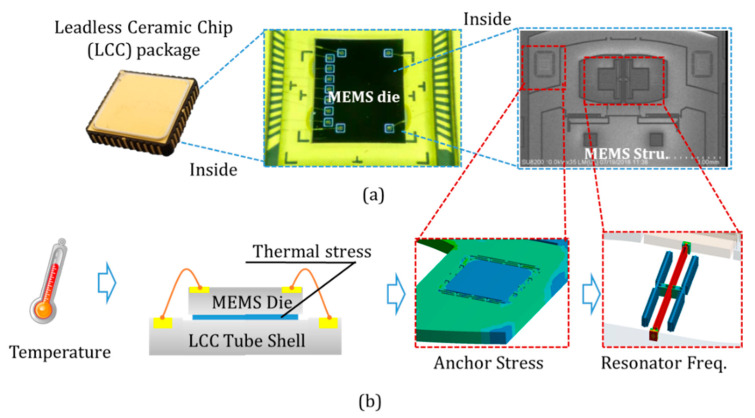
(**a**) Leadless Ceramic Chip (LCC) package with MEMS chip; (**b**) The mechanism of resonator frequency shift due to temperature.

All these efforts significantly improve the temperature coefficient of the silicon resonators. However, the frequency and temperature characteristics of the resonator are also affected by the packaging method. Packaging processes bring different materials, leading to intrinsic stress with different temperatures. The Leadless Ceramic Chip (LCC) package chip is made of ceramic materials. The silicon dies, containing a structural layer and a substrate layer, is bonded to the shell by adhesive resistance, as illustrated in Figure 1. The adhesive is generally cured at high temperatures. Its thermal expansion coefficient (TEC) is quite different from that of silicon and ceramic materials [17]. The resonator’s stress is produced and varied during cooling to operating temperature, ranging from −40 °C to 60 °C. Internal stress changes the resonator’s natural frequency, leading to the vibration frequency varies with temperature.

The temperature coefficient of the resonator comes from two parts. The one is the material intrinsic frequency variations with the temperature. The other one is the change of supporting environment, which served as boundary conditions in the mechanical model. The variations of pre-stress lead to the frequency drift due to temperature. The thermal stress during the adhesion process is introduced to study the frequency drift law of the resonator. The four-layer model was established, consists of a ceramic shell, adhesive resist, silicon handle wafer, and silicon device layer. The resonator frequency variations with temperature under different support architectures were calculated and analyzed. The resonant beam accelerometer was used in the experiments to verify the theoretical model. Different adhesive resists were implemented in the packaging of the accelerometer chip and ceramic shell. We also changed the bonding processes to find the optimized packaging method with the minimum temperature coefficient. The results indicate that the optimal packaging method achieves the −24.1 ppm/°C to −30.2 ppm/°C temperature coefficient of the resonator, close to the intrinsic value of silicon (−31 ppm/K).

## 2. Materials and Methods

### 2.1. Resonator Frequency and Intrinsic Stress

**Figure 2:**
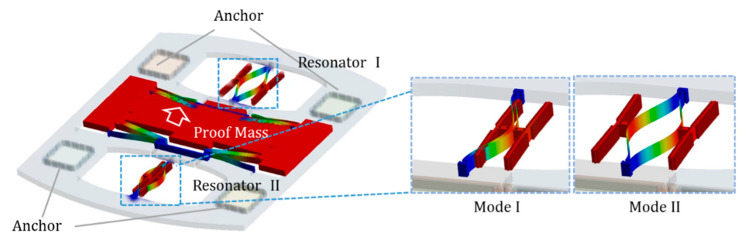
Operating principle of resonant accelerometer with two resonators.

The resonant accelerometer is a typical MEMS device that consists of a proof mass, two resonant beams with capacitive combs, flexural beams, a frame, and anchors [18,19]. The resonator has two modes with the movement in the same direction or the opposite direction. The mode I illustrated in Figure 2 is symmetric motion, where two resonant beams move in the same direction. Mode II is the antisymmetric mode, and the two vibrant beams exhibit opposite movements. The symmetric motion is the operating mode of the accelerometer. The capacitive combs provide electrostatic driving forces and displacement detection as well as suspension mass of the resonance beam. The proof mass generates displacement along the axial direction when external acceleration is input. The displacement is transferred through levers and comes into being compressive and tensile stress of resonate beams to change the frequency. So, the accelerometer has differential resonators that effectively resist common-mode interference. It is an ideal candidate for the temperature characteristics of the resonator.

**Figure 3:**
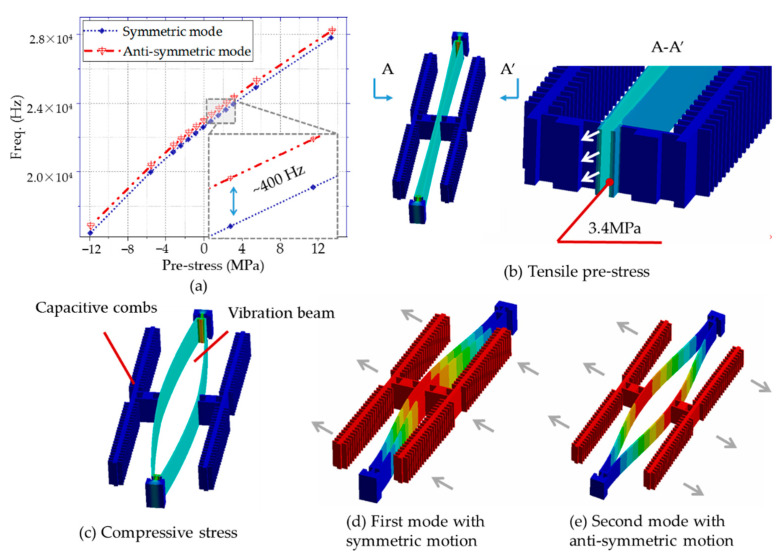
(**a**) The relationship between pre–stress and resonate frequencies; (**b**) Strain and stress distributions on the cross–section of the resonant beam under tensile stress; (**c**) Strain and stress under compressive stress; (**d**,**e**) Symmetric and antisymmetric motion of resonance beam.

Figure 3 indicates the relationship between pre-stress and resonate frequencies through the finite element analysis. The symmetric mode frequency is about 400 Hz lower than the antisymmetric modes, located in the first order. Resonator frequency varies with internal stress, which decreases under compressive stress and increases under tensile stress. Strains occur in the structure due to the difference in thermal expansion coefficients of materials, resulting in stress, which leads to the change of resonator frequency [10]. Thermomechanical stresses induced by the packaging assembly processes are complicated, and different packaging causes various temperature coefficients [20]. 

### 2.2. The Model of the Resonator Temperature Characteristic

**Figure 4:**
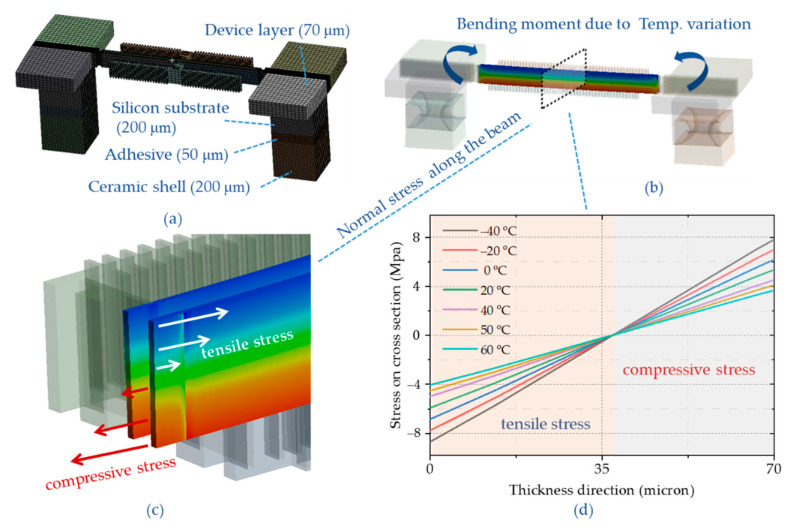
(**a**) The double-clamped resonator model for temperature characteristic; (**b**) Bending moment deformation caused by thermal stress; (**c**) Distribution of axial normal stress on cross-section profile; (**d**) The normal stress distribution along the longitudinal direction of the beam with different temperature.

Four-layer structure models were established to address the resonator temperature characteristics due to thermal expansion coefficient mismatch [21]. There are ceramic shell, adhesive layer, silicon handle layer, and silicon device layer. As shown in Figure 4, the anchors are mounted in the axial direction of the resonant beam. The resonator produces a bending moment at operating temperature due to temperature variation. 

**Figure 5:**
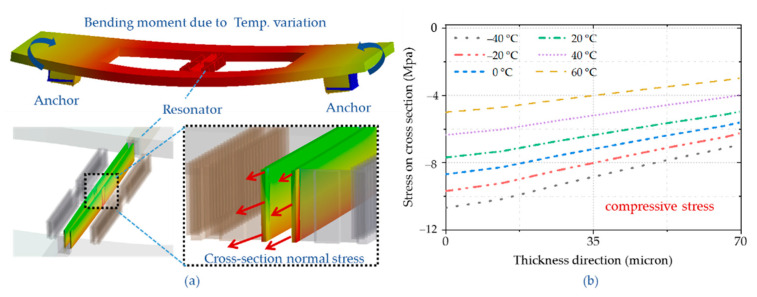
(**a**) Resonator model mounted by frame and the axial normal stress distributions; (**b**) The normal stress distribution along the longitudinal direction of the beam with different temperatures.

The normal stress distributions on cross-section are according to the bending moment’s characteristics. The tensile stress is above the neutral axis, and the compressive stress is below the neutral axis. The stress changes linearly along the longitudinal direction of the beam, from tensile stress to compressive stress. The bending moment decreases with the rising of temperature, as illustrated in Figure 4d. The axial normal stress of the resonance beam varies depending on the fastening architectures. The resonator is mounted by a frame, where anchors are located on both sides of the resonance beam (Figure 5). Bending moments are generated between two anchors due to the thermal stress. The normal stress distribution along the longitudinal direction of the beam is compressive stress with decreasing amplitude. 

This paper selected two adhesive types to determine the influence of different adhesive properties on packaging stress and frequency characteristics. The hard adhesive is the material with a higher Young’s modulus. As listed in Table 1, the ABP 84-3JT adhesive from LocTite was used in the experiments and calculations. The modulus is 2.95 GPa below25 °C and 0.589 GPa above 150 °C. The soft adhesive employed in the paper is a silicone adhesive with a lower Young’s modulus, which is 5 MPa in solid-state. TEC among materials varies greatly, where TEC of hard adhesive is about 300 ppm/K linearly. The TEC of silicon is 2 ppm/K, nearly two orders of magnitude lower than adhesive. 

**Figure 6:**
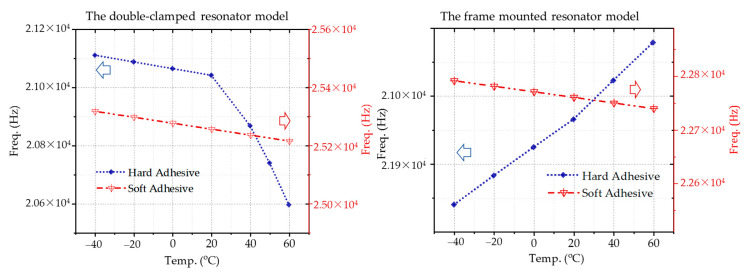
The resonator temperature characteristic with different adhesive in two models.

The stress distribution analyzed above shows that the resonator temperature characteristic with different adhesive in two models is illustrated in Figure 6. The temperature coefficient is expressed as,

(1) $$\alpha =\frac{f_{i+1}-f_{i}}{\left(T_{i+1}-T_{i}\right)f_{i}}$$

where *f_i_* is the resonator frequency at the temperature *T_i_*. In the double-clamped resonator model, the frequency decreases with the rising temperature for both hard or soft adhesive. The temperature coefficient utilizing hard adhesive is from −23 ppm to −174 ppm, which is larger than that with soft adhesive (about −40 ppm within the temperature range). The situation is different for the frame-mounted model. The temperature coefficient utilizing hard adhesive is positive, while that of soft adhesive is negative. The compressive stress is dominant and released with the increase of temperature, resulting in the rising frequency.

### 2.3. The Modeling and Experiments of the Resonant Accelerometer

**Figure 7:**
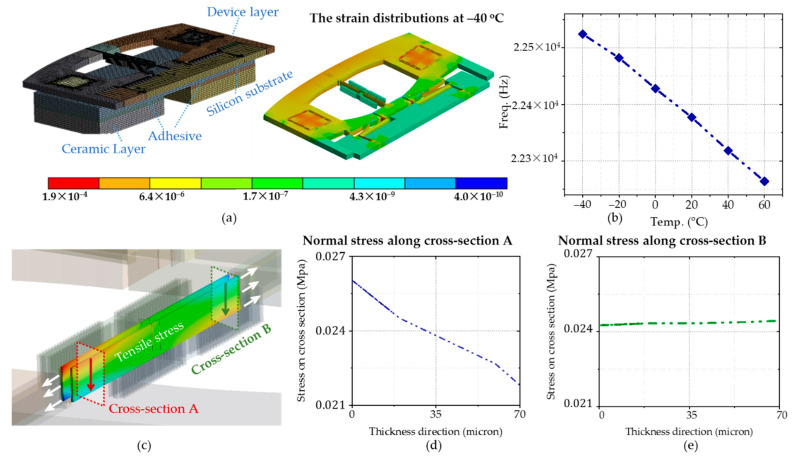
(**a**) The finite element model of the Resonant accelerometer and the train distributions under −40 °C with hard adhesive; (**b**) The relationship between temperature and symmetric motion resonate frequency; (**c**) The resonance beam exhibits tensile stress in operating temperature; (**d**,**e**) Normal stress along with cross-section A & B.

The finite element model of the Resonant accelerometer was established to study the stress and temperature characteristics of the resonator. Considering that the structure is symmetrical, half of the accelerometer is modeled to reduce the calculation amount. The frame sags to the center, and the stress distribution of the resonance beam is asymmetrically bonded with hard adhesive at −40 °C, as illustrated in Figure 7. The stress decreases from 26.5 kPa to 21 kPa from top to bottom in cross-section A, and the value is maintained at 24 kPa in cross-section B. Both of them are tensile stress. The stress inside the resonate beam determines the resonate frequency. The structure’s frequency temperature characteristic is obtained by calculating the models of different temperature points from −40 °C to 60 °C.

**Figure 8:**
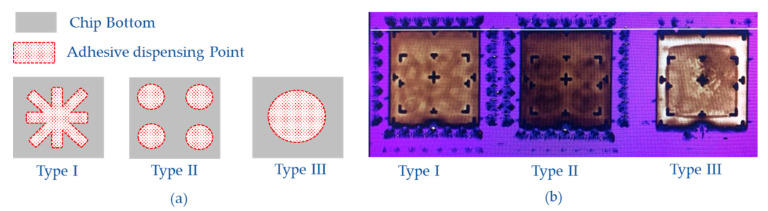
(**a**) Three types of adhesive dispensing method; (**b**) The photography of chips bottom with different adhesive dispensing ways.

Three types of adhesive dispensing methods were taken place in the experiments to optimize the bonding method, as shown in Figure 8. The fastening center and eight radiation directions are confirmed by the double-cross pattern shown in the first dispensing type to achieve good shear strength. The second type fixes the four corners of the chip. A center dispensing point constructs the third pattern. After packaging with different types, the temperature experiments were carried out every 10 °C in the range of −40 °C to 60 °C. Each temperature point was kept for 2 h to reach the equilibrium state, and then the resonate frequencies were tested.

## 3. Results

Temperature characteristic tests of resonant accelerometers with different bonding methods and adhesives were taken place every 10 °C as listed in Table 2 and Table 3. The frequency decreases with the rising of the ambient temperature. The frequency of resonators with hard adhesive is higher than that with a soft one. The frequency temperature coefficient is obtained by the frequency difference ratio to temperature variation multiplied by frequency value. The type III fixed pattern samples’ amplitude is slightly larger than that of Type I and II. 

**Figure 9:**
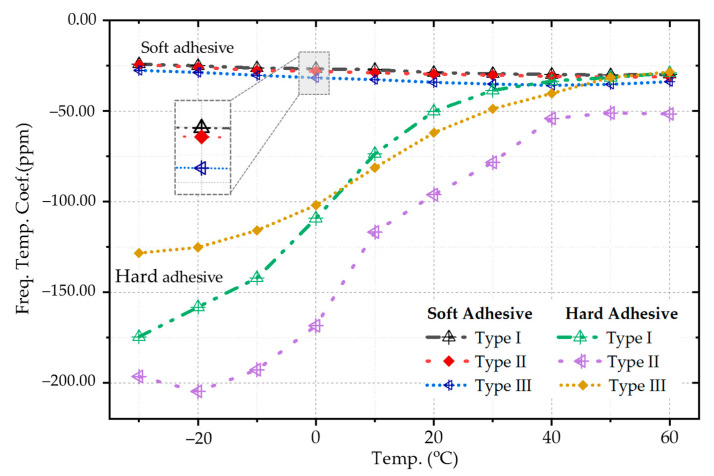
The frequency temperature coefficient in the test with hard & soft adhesive in three dispensing methods.

The frequency decreases with the increase of temperature for all samples, which means that the temperature coefficient is negative. Figure 9 indicates the comparison in various situations. The samples’ temperature coefficient with soft adhesive, which has a smaller Young’s modulus, is lower than the samples with hard adhesive in the whole temperature range. The temperature coefficients of the three packaging methods have little difference with soft adhesive bonding. The high-temperature data is slightly higher than that of low temperature, but the difference is within 6 ppm. The samples with Type I dispensing method achieve −24.1 ppm/°C to −30.2 ppm/°C, which is also the best one among three packaging ways. The performance of the samples with type II adhesive dispensing process is close to that of with Type I, where the temperature coefficient is from −24.5 ppm/°C to −31.2 ppm/°C. The samples with type III packaging process have a slightly higher temperature coefficient in −27.6 ppm/°C to −35.8 ppm/°C. 

The situation is different with hard adhesive. The general law is that the temperature coefficient is more extensive at low temperatures and decreases gradually with the increase of temperature. The chips with four corners bonding are worst, which means sizeable normal stress occurs in the resonant beams. In this case, the chip is fully constrained, and the strain caused by the mismatch of TEC is transferred to the resonant beams, resulting in a larger frequency gradient. The central fixed chip achieves better performance. The temperature coefficient is about −128.4 ppm/°C at −30 °C, and in the high-temperature region, the value is −28.5 ppm/°C. The chips with a double-cross bonding pattern (Type I) has better performance above 10 °C. The resonator frequency arises faster with the reduction of temperature below 0 °C. Both packaging methods release thermal stress to decrease the temperature coefficient.

**Figure 10:**
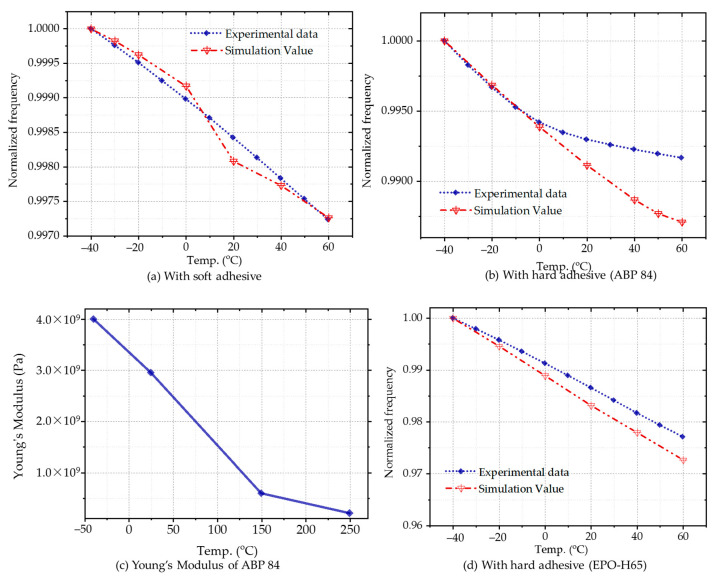
Comparing temperature characteristics between experimental and simulation results, (**a**) the data with soft adhesive; (**b**) the data with hard adhesive ABP 84-3JT; (**c**) Young’s Modulus of ABP 84-3JT applied in the simulation; (**d**) the data with hard adhesive EPO-H65.

The comparison of experimental and simulation results was illustrated in Figure 10. The frequency at each temperature point is normalized due to silicon’s Young’s modulus between the simulation and the real value. The frequency variation of the resonator can be studied more clearly by the relative value. All of the data were implemented utilizing Type II dispensing pattern. During the packaging process with soft adhesive, SemiCosil 989(8)/1 K silicone adhesive was selected. The frequency varies by 0.275% in the temperature range of 100 °C, consistent with experimental results. In terms of hard adhesive packaging, ABP 84-3JT and EPO-H65 were used in the experiments. The frequency gradient at a lower temperature is more extensive than at a high temperature for ABP 84-3JT. The Young’s Modulus of the adhesive (shown in Figure 10c) significantly affects the frequency variation trend. Compared with the experimental results, the simulation value exhibits better consistency below 0 °C. A slight nonlinearity was observed above 40 °C, but the inflection point appears at 0 °C in the experiment. The experimental results are in better agreement with the simulation value with EPO-H65, Young’s modulus of which is 916,396 psi (equals 6.318 GPa), higher than that of ABP 84-3JT. In comparing two hard adhesives, the frequency variation is more extensive for EPO-H65. The temperature coefficient is about −200 ppm/°C in the whole temperature range. The samples with ABP 84-3JT exhibit a variable temperature coefficient, which is higher below 0 °C.

## 4. Conclusions

The paper investigates the frequency drift laws caused by the thermal stress in the packaging process. The four-layer model was established to discover the stress distributions in different conditions. The frequency decreases with the rising temperature for both hard or soft adhesive within the double-clamped model. The temperature coefficient utilizing hard adhesive is positive, while that of soft adhesive is negative. The resonant accelerometer was employed for the frequency measurements. Temperature characteristic tests with different bonding methods and adhesives were taken place every 10 °C. Three types of adhesive dispensing methods mean various chip constraints. The results indicate that the frequency varies about −150 ppm/°C at low temperatures. The amplitude of the frequency gradient decreases as the rising temperature with the hard adhesive. The temperature coefficient employing soft adhesive, which has a smaller Young’s modulus, is lower in the whole temperature range. The optimal packaging method achieves −24.1 ppm/°C to −30.2 ppm/°C temperature coefficient of the resonator in the whole temperature range, close to the intrinsic property of silicon (−31 ppm/°C).

## Figures and Tables

**Table 1:** The material parameters applied in the calculation.

Material	Density (kg/m^3^)	Young’s Modulus (MPa)	Poisson’s Ratio	Thermal Conductivity(W/m∙K)	Coefficient of Thermal Expansion (ppm/K)
Silicon	2330	1.69 × 10^11^	0.27	130	2
Ceramics	3970	3.1 × 10^11^	0.28	14	7.1
Hard Adhesive	1500	2.95 × 10^9^/5.89 × 10^8 (1)^	0.3	0.6	63/117 ^(2)^
Soft Adhesive	1100	5 × 10^6^	0.3	0.8	300

^(1)^ The Modulus at 25 °C is 2950 MPa and 589 MPa at 150 °C. ^(2)^ The data below glass transition temperature (56 °C)/above transition temperature.

**Table 2:** The temperature characteristic test results of the resonator with soft adhesive.

Temp. (°C)	Freq. (Hz)			Freq. Diff. (Hz)		Freq. Temp. Coef. (ppm/°C)			
	Type I	Type II	Type III	Type I	Type II	Type III	Type I	Type II	Type III
−40	24,891.2	24,835.4	25,148.4	-	-	-	-	-	-
−30	24,885.2	24,829.3	25,141.5	−6.0	−6.1	−6.9	−24.1	−24.5	−27.6
−20	24,878.9	24,822.8	25,134.3	−6.2	−6.4	−7.2	−25.1	−25.9	−28.7
−10	24,872.4	24,816.0	25,126.7	−6.5	−6.9	−7.6	−26.3	−27.7	−30.3
0	24,865.7	24,809.0	25,118.7	−6.6	−6.9	−8.0	−26.7	−28.0	−31.7
10	24,859.0	24,801.9	25,110.5	−6.8	−7.2	−8.2	−27.2	−28.9	−32.7
20	24,851.8	24,794.5	25,101.9	−7.2	−7.4	−8.6	−28.8	−29.7	−34.2
30	24,844.5	24,787.0	25,093.1	−7.3	−7.5	−8.8	−29.4	−30.2	−35.1
40	24,837.1	24,779.4	25,084.1	−7.4	−7.7	−9.0	−29.9	−30.9	−35.8
50	24,829.6	24,771.6	25,075.3	−7.5	−7.7	−8.8	−30.2	−31.2	−35.3
60	24,822.2	24,763.9	25,066.8	−7.4	−7.7	−8.5	−29.8	−31.1	−33.8

**Table 3:** The temperature characteristic test results of the resonator with hard adhesive (ABP 84-3JT).

Temp. (°C)	Freq. (Hz)			Freq. Diff. (Hz)			Freq. Temp. Coef. (ppm/°C)		
	Type I	Type II	Type III	Type I	Type II	Type III	Type I	Type II	Type III
−40	25,523.0	25,584.3	25,169.6	−	−	−	−	−	−
−30	25,478.5	25,534.2	25,137.3	−44.5	−50.2	−32.3	−174.6	−196.5	−128.4
−20	25,438.3	25,482.0	25,105.9	−40.2	−52.2	−31.4	−158.2	−204.7	−125.2
−10	25,402.2	25,433.0	25,076.8	−36.1	−49.0	−29.1	−142.1	−192.8	−115.9
0	25,374.5	25,390.2	25,051.3	−27.7	−42.8	−25.6	−109.2	−168.5	−102.0
10	25,355.8	25,360.6	25,030.9	−18.7	−29.6	−20.3	−73.6	−116.8	−81.3
20	25,343.1	25,336.2	25,015.4	−12.7	−24.4	−15.5	−50.2	−96.2	−62.0
30	25,333.3	25,316.4	25,003.2	−9.8	−19.9	−12.2	−38.6	−78.4	−48.8
40	25,324.8	25,302.7	24,993.1	−8.5	−13.7	−10.1	−33.5	−54.1	−40.3
50	25,316.8	25,289.7	24,985.3	−8.0	−12.9	−7.9	−31.5	−51.1	−31.5
60	25,309.5	25,276.7	24,978.1	−7.3	−13.0	−7.1	−29.0	−51.6	−28.5
